# Gut microbiota and polycystic ovary syndrome, focus on genetic associations: a bidirectional Mendelian randomization study

**DOI:** 10.3389/fendo.2024.1275419

**Published:** 2024-01-22

**Authors:** Jing Wang, Pier Luigi Fiori, Giampiero Capobianco, Ciriaco Carru, Zhichao Chen

**Affiliations:** ^1^ Department of Obstetrics and Gynecology, Second Affiliated Hospital of Shantou University Medical College, Shantou, China; ^2^ Department of Biomedical Sciences, University of Sassari, Sassari, Italy; ^3^ Department of Cardiology, Second Affiliated Hospital of Shantou University Medical College, Shantou, China

**Keywords:** gut microbiota, causality, polycystic ovary syndrome (PCOS), Mendelian randomization, genetic

## Abstract

**Background:**

The contribution of gut microbiota to the pathogenesis of polycystic ovary syndrome (PCOS) is controversial. The causal relationship to this question is worth an in-depth comprehensive of known single nucleotide polymorphisms associated with gut microbiota.

**Methods:**

We conducted bidirectional Mendelian randomization (MR) utilizing instrumental variables associated with gut microbiota (N = 18,340) from MiBioGen GWAS to assess their impact on PCOS risk in the FinnGen GWAS (27,943 PCOS cases and 162,936 controls). Two-sample MR using inverse variance weighting (IVW) was undertaken, followed by the weighted median, weighted mode, and MR-Egger regression. In a subsample, we replicated our findings using the meta-analysis PCOS consortium (10,074 cases and 103,164 controls) from European ancestry.

**Results:**

IVWMR results suggested that six gut microbiota were causally associated with PCOS features. After adjusting BMI, SHBG, fasting insulin, testosterone, and alcohol intake frequency, the effect sizes were significantly reduced. Reverse MR analysis revealed that the effects of PCOS features on 13 gut microbiota no longer remained significant after sensitivity analysis and Bonferroni corrections. MR replication analysis was consistent and the results suggest that gut microbiota was likely not an independent cause of PCOS.

**Conclusion:**

Our findings did not support the causal relationships between the gut microbiota and PCOS features at the genetic level. More comprehensive genome-wide association studies of the gut microbiota and PCOS are warranted to confirm their genetic relationship.

**Declaration:**

This study contains 3533 words, 0 tables, and six figures in the text as well as night supplementary files and 0 supplementary figures in the Supplementary material.

## 1 Introduction

Polycystic ovary syndrome (PCOS) is a common endocrine disorder impacting reproduction and metabolism in women of reproductive age. Although PCOS is a heterogeneous and multi-phenotype syndrome, it is mostly characterized by hyperandrogenism, anovulation, and polycystic ovary morphology (1, 2). PCOS affects 5–18% of reproductive-aged women (3, 4), with severe health conditions, leading to infertility, miscarriage, and various pregnancy complications, as well as increased risk of endometrial cancer, cardiovascular disease, type 2 diabetes, depression, and anxiety (5–11). However, the complex pathogenic mechanisms, such as heredity, hypothalamic and ovarian dysfunction, and insulin resistance, result in unclear etiology, suboptimal treatment outcomes, and healthcare-related economic burdens (3, 12).

Emerging studies have implicated gut microbiota in the pathogenesis of PCOS (13–15). The gut microbiota is a complex ecosystem of archaea, bacteria, fungi, viruses, and protozoa (16). It plays essential functions for the human body in metabolism, immunity, and nervous system (17). For instance, the gut microbiota is involved in developing cardiovascular disease, type 2 diabetes, and neurodegenerative diseases (18–20). Previous studies show that the gut microbiota may impact the onset and progression of PCOS through the endotoxemia pathway (21), the gut-brain axis (22), the gut microbiota-bile acid-interleukin-22 axis (23), and other pathways (24). Furthermore, the treatment of utilizing fecal microbiota transplants of healthy rats to the PCOS ones resulted in reduced androgen biosynthesis and normalized ovarian morphology (15).

Most studies on the association between the gut microbiota and PCOS are observational, with results susceptible to confounding bias (e.g., region, diet). In contrast, Mendelian randomization (MR) is an efficient approach to avoid confounding bias by utilizing genetic variants related to exposure factors as instrumental variables for evaluating the association of exposure factors with disease (25). MR analysis has been extensively conducted to investigate causal relationships between gut microbiota and diseases, such as the associations between gut microbiota and systemic lupus erythematosus, depression, and cardiometabolic disease (26–28).

In the present study, we undertook a bidirectional two-sample MR research utilizing summary statistics of genome-wide association studies (GWAS) to explore genetic associations between gut microbiota and PCOS features.

## 2 Materials and methods

**Figure 1:**
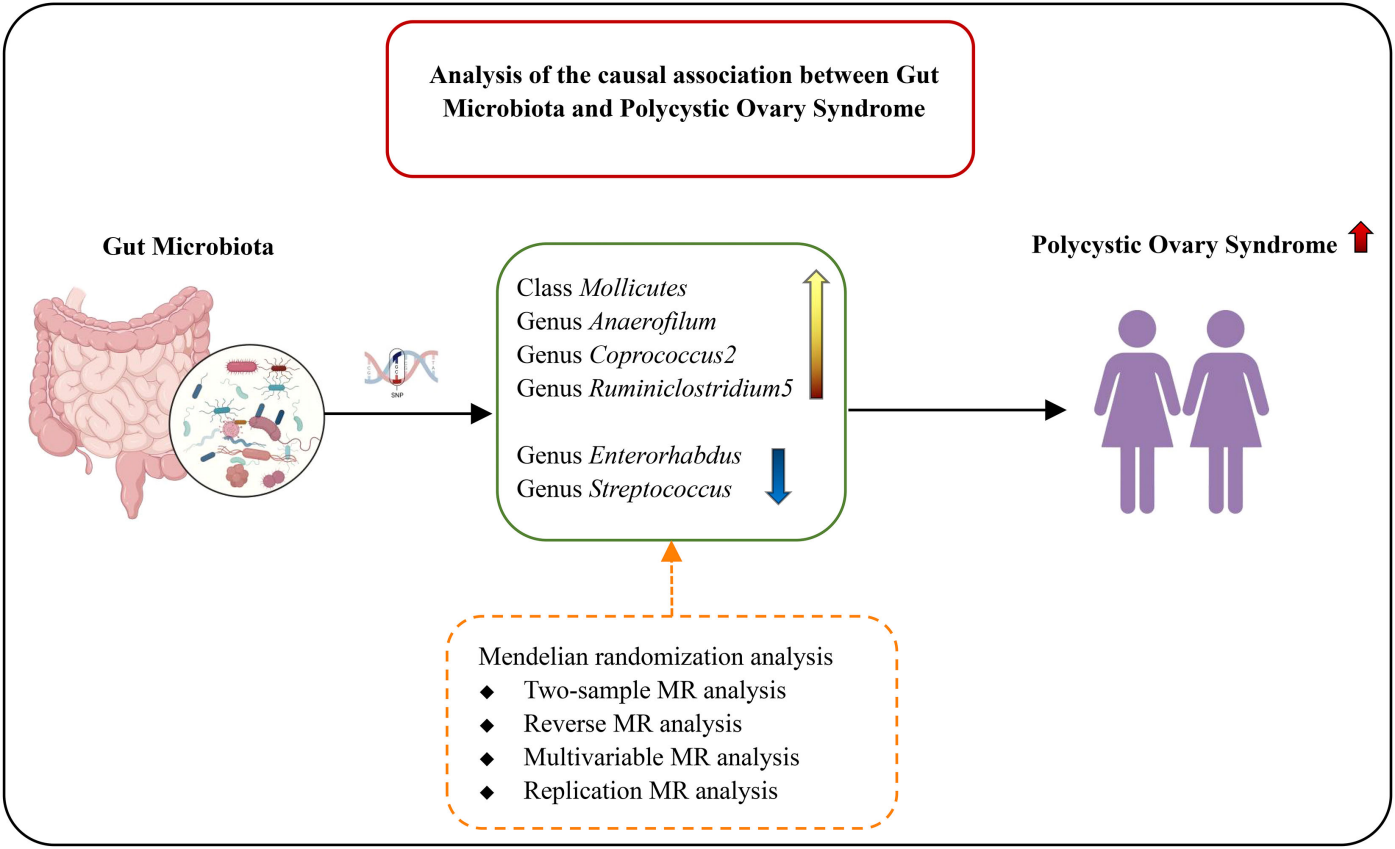
The flow chart of the study.

We complied with the Strengthening Reporting of Observational Studies in Epidemiology Using Mendelian Randomization Methods (STROBE-MR) to implement this study (29), as shown in **Supplementary Table 1**. We first assessed the genetic correlation between exposure and outcome. Second, we conducted a bidirectional two-sample MR analysis. Third, we coordinated potential risk factors for multivariate MR analysis. In addition, we performed replication analyses using another PCOS database. The flow chart of the study is in **Figure 1**.

### 2.1 Study population

Genetic variations of the gut microbiota abundance were obtained from the MiBioGen, the enormous-scale and multi-ethnic study of human gut microbiota genetics to date (30). The research included genome-wide genotype and 16S fecal microbiota data analyzed for 18,340 participants in 24 cohorts, most from Europe (N = 13,266). There are 211 taxa in the human gut microbiota, including 12 unknown genera and three unknown families. Thus, we analyzed 196 known taxa.

Extracted outcome PCOS GWAS statistics from FinnGen Consortium R8 release data (31). This research analyzed 20,175,454 variants from 342,499 individuals. A total of 27,943 PCOS cases and 162,936 controls were included in the analysis after adjusting for sex, age, and the first ten principal components. The diagnosis of PCOS was according to the International Classification of Diseases (ICD) 8th Revision, ICD 9 and ICD 10. Patients presented primarily with hyperandrogenemia and ovarian dysfunction. The replication analyses of the PCOS were derived from 10,074 cases and 103,164 controls of European ancestry, with diagnoses based on National Institutes of Health (NIH), or Rotterdam criteria, or self-report (32).

As the gut microbiota-disease relationships were influenced by major confounders (33) as well as obesity, hyperandrogenism, and insulin resistance were associated with the onset and progression of PCOS (3). Prior research suggested that gut microbiota may influence the development of PCOS through sex hormone regulation (34). We undertook multivariable MR analyses with obesity, sex hormone-binding globulin (SHBG), alcohol intake frequency, fasting insulin, and total testosterone as confounders. A body mass index (BMI) of 30 kg/m^2^ or more is considered obesity (35). We obtained genetic variations associated with BMI (N = 461,460), alcohol intake frequency (N = 462,346), and total testosterone(N = 199,569) from MRC-IEU. Genetic variations related to SHBG (N = 214,989) were extracted from the UK Biobank. In addition, We extracted genetic variation associated with fasting insulin from the Meta-Analyses of Glucose and Insulin-related Traits Consortium (MAGIC). The sample population information in this study is presented in **Supplementary Table 2**.

### 2.2 Genetic instrumental variables

Single nucleotide polymorphisms (SNPs) are used as instrumental variables (IVs) in MR analysis. To ensure the robustness of the results, we selected the optimal IVs to analyze according to the following steps. First, we extracted SNPs related to gut microbiota based on genome-wide significance (*P* < 5 × 10^-8^). Since the number of qualified SNPs was too small, we extended the threshold to *P* < 1 × 10^-5^ for more comprehensive causalities. Second, we analyzed the linkage disequilibrium (LD) between SNPs (r^2^ < 0.001, clumping distance = 10,000kb) and excluded unqualified SNPs. We also examined the selected IVs on PhenoScanner (http://www.phenoscanner.medschl.cam.ac.uk) and manually removed SNPs associated with potentially confounding factors. Finally, we harmonized exposure and outcome SNPs and deleted the palindromic SNPs to maintain the consistency of effect alleles.

To assess the strength of IVs, we calculated the F statistic of each SNP by using the formula: *F* = β^2^/σ^2^ (β and σ represent the effect estimate and standard deviation (SD) of the exposure SNP, respectively). The IV was considered strong enough (*F* statistic > 10) to avoid the effects of weak instrumental bias in the results of the MR analyses (36). In addition, we calculated *R* ^2^ to indicate the proportion of variance explained by the association between the SNP and the exposure variable. This was calculated as *R* ^2 =^ 2 × (1 – EAF) × EAF × β^2^, where EAF is the effective allele frequency.

### 2.3 Statistical analysis

We conducted all statistical analyses in R software (version 4.2.2) using the packages “MendelianRandomization,” “TwoSampleMR,” “MVMR,” and “GenomicSE”. The odds ratio (OR) with a 95% confidence interval (CI) indicated estimates, with *P* < 0.05 regarded as a statistically significant result. We applied the Bonferroni multiple testing correction adjusted threshold of *P* < 2.55 × 10^-4^ (0.05/196) to identify statistically significant causal relationships (37).

#### 2.3.1 MR analysis

We performed cross-trait linkage disequilibrium score (LDSC) regression to compute the genetic correlation between gut microbiota and PCOS (38). The inverse variance weighted (IVW) test with the random effects was performed as the main MR method to calculate the causal effect of gut microbiota and PCOS (39). The effect values of individual SNP were computed by the Wald ratio method. Accounting for pleiotropy, we applied three additional MR models: weighted median (40), weighted mode (41), and MR-Egger regression (42). The weighted median approach contributed to data analysis with 50% of the genetic variation from invalid IVs and still provided a consistent causal effect estimate. The MR-Egger relies on the genetic instrument strength independence of direct effects (InSIDE) assumption and the NO measurement error (NOME) assumption. We considered the results robust when the causal effects of the four MR methods were consistent. Subsequently, we performed a replication analysis of the data from another PCOS sample to ensure the reliability of the results.

#### 2.3.2 Sensitivity analysis

We conducted Cochran’s *Q* test for heterogeneity analysis, and a *P* less than 0.05 was considered heterogeneity (43). The MR-Egger intercept test and Randomization Pleiotropy Residual Sum and Outlier (MR-PRESSO) global test were performed to detect horizontal pleiotropy (42, 44). To evaluate the direction of potential causalities, we used the MR Steiger filtering method (45). In addition, we used the “leave-one-out” analysis to examine the stability of our results.

Since studies on relationships between gut microbiota and disease could be influenced by confounding factors such as lifestyle, there is a risk of false-positive causality (33). Obesity (BMI), alcohol intake frequency, SHBG, total testosterone, and fasting insulin levels affect the host’s gut microbiota and are also associated with the risk of PCOS. Therefore, we conducted a multivariate MR analysis with them as covariates to minimize the effect of confounders on the results.

#### 2.3.3 Reverse MR analysis

To investigate the causal effect of PCOS features on gut microbiota, we performed reverse MR using PCOS as the exposure factor and gut microbiota as the outcome.

## 3 Results

### 3.1 Genetic instrumental variables

A total of 2,037 SNPs were screened for IV (*P* < 1 × 10^-5^) based on rigorous criteria, including nine phyla (103 SNPs), 15 classes (180 SNPs), 20 orders (218 SNPs), 30 families (341 SNPs), and 122 genera (1195 SNPs). Each SNP showed sufficient strength as all *F* statistics were greater than 10 (from 16.83 to 88.83). The *R* ^2^ for the proportion of variance explained by the association between each SNP and exposure variable ranged from 0.07% to 1.01%. Details of all SNPs are displayed in **Supplementary Data S1**.

According to the genome-wide significance threshold of *P* < 5 × 10^-8^, only 21 SNPs were significantly associated with gut microbiota. These included one phylum (1 SNP), one class (1 SNP), two orders (1 SNP), four families (5 SNPs), and ten genera (11 SNPs). There were no weak instrumental variables (*F* statistics from 29.51 to 88.83). The *R* ^2^ for each SNP ranged from 0.18% to 1.01%. Information on all SNPs is provided in **Supplementary Data S2**.

### 3.2 MR analysis (locus-wide significance, *P<*1×10^-5^)

**Figure 2:**
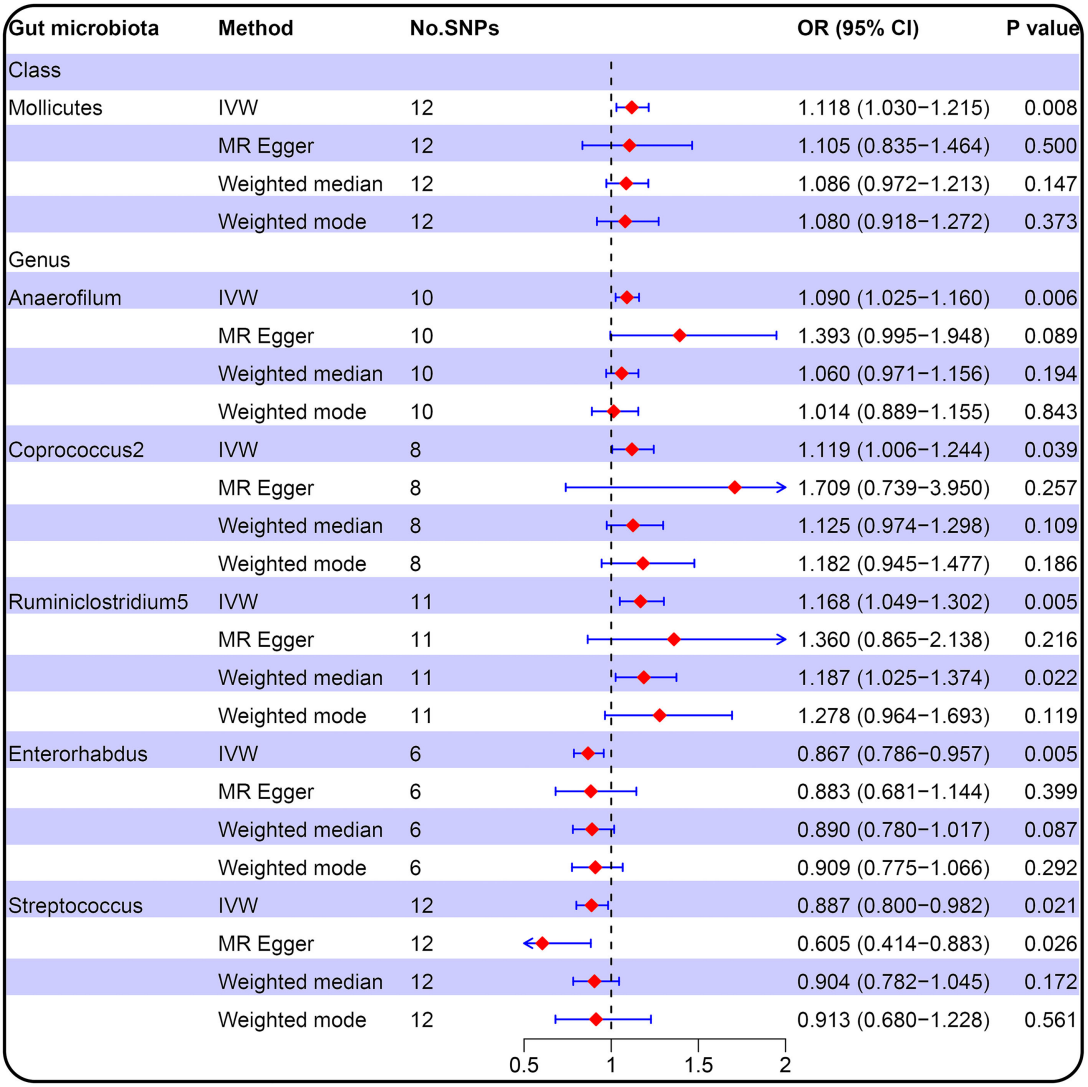
Forest plot of the causal relationship between gut microbiota and PCOS (locus-wide significance, P<1×10^-5^).

We conducted MR analysis on the association of 196 gut microbial abundances and PCOS features (detailed results in **Supplementary Data S4**), and one class and five genera passed the significance threshold of 0.05 (**Figure 2**; **Supplementary Table 4**). We queried the SNPs used for the above statistically significant causality analyses on Phenoscanner, which revealed no SNPs with confounding effects (**Supplementary Data S5**). LDSC regression analysis for statistically significant causal associations between six gut microbiota taxa and PCOS suggested that only genus *Anaerofilum* was genetically correlated with PCOS (rg = 0.724, *P* = 0.024). In contrast, the rest were not (**Supplementary Table 3**).

**Figure 3:**
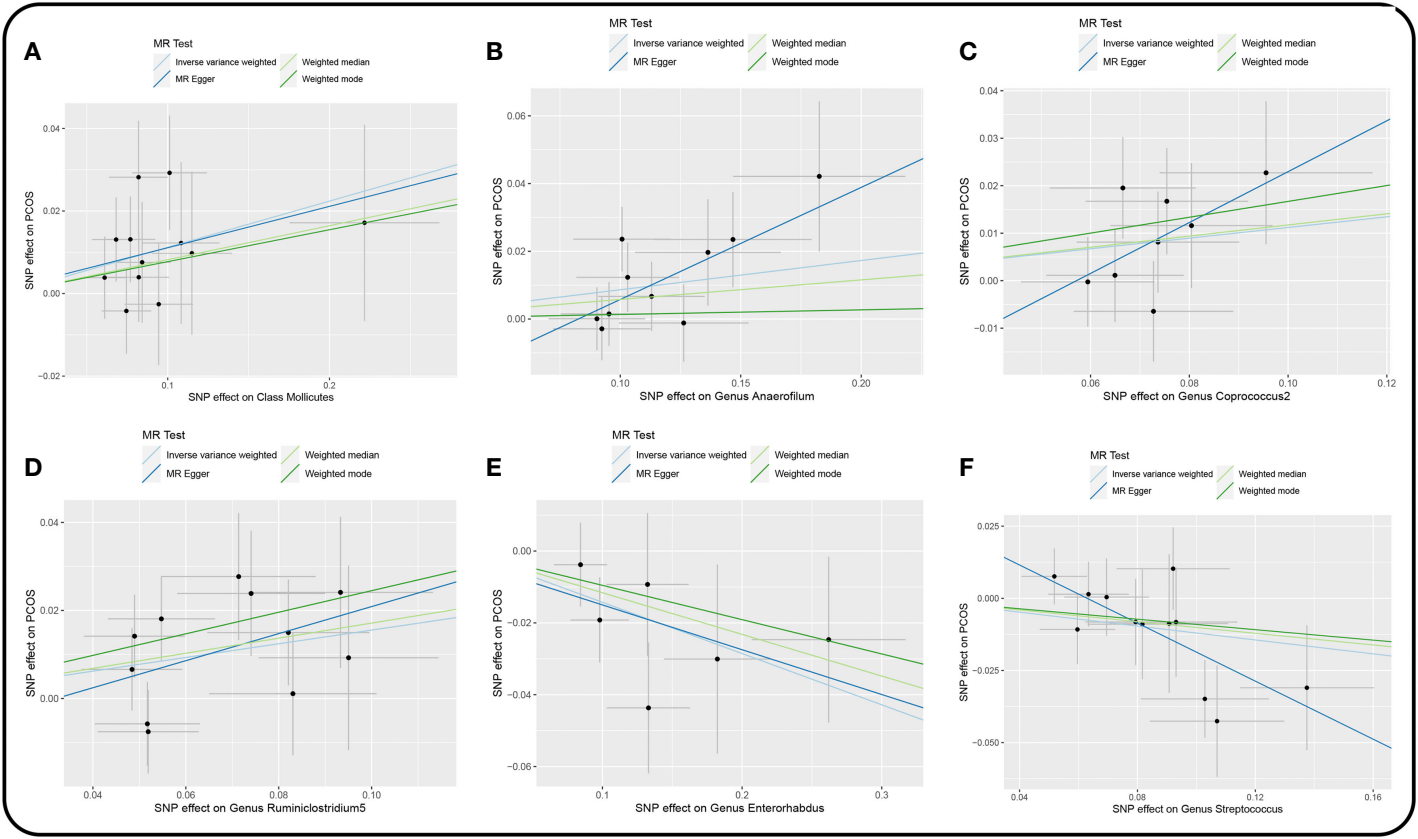
Scatter plot of the univariable MR analysis of gut microbiota and PCOS (locus-wide significance, P<1×10^-5^). **(A)** Class *Mollicutes*. **(B)** Genus *Anaerofilum*. **(C)** Genus *Coprococcus*2. **(D)** Genus *Ruminiclostridium*5. **(E)** Genus *Enterorhabdus*. **(F)** Genus *Streptococcus*.

**Figure 4:**
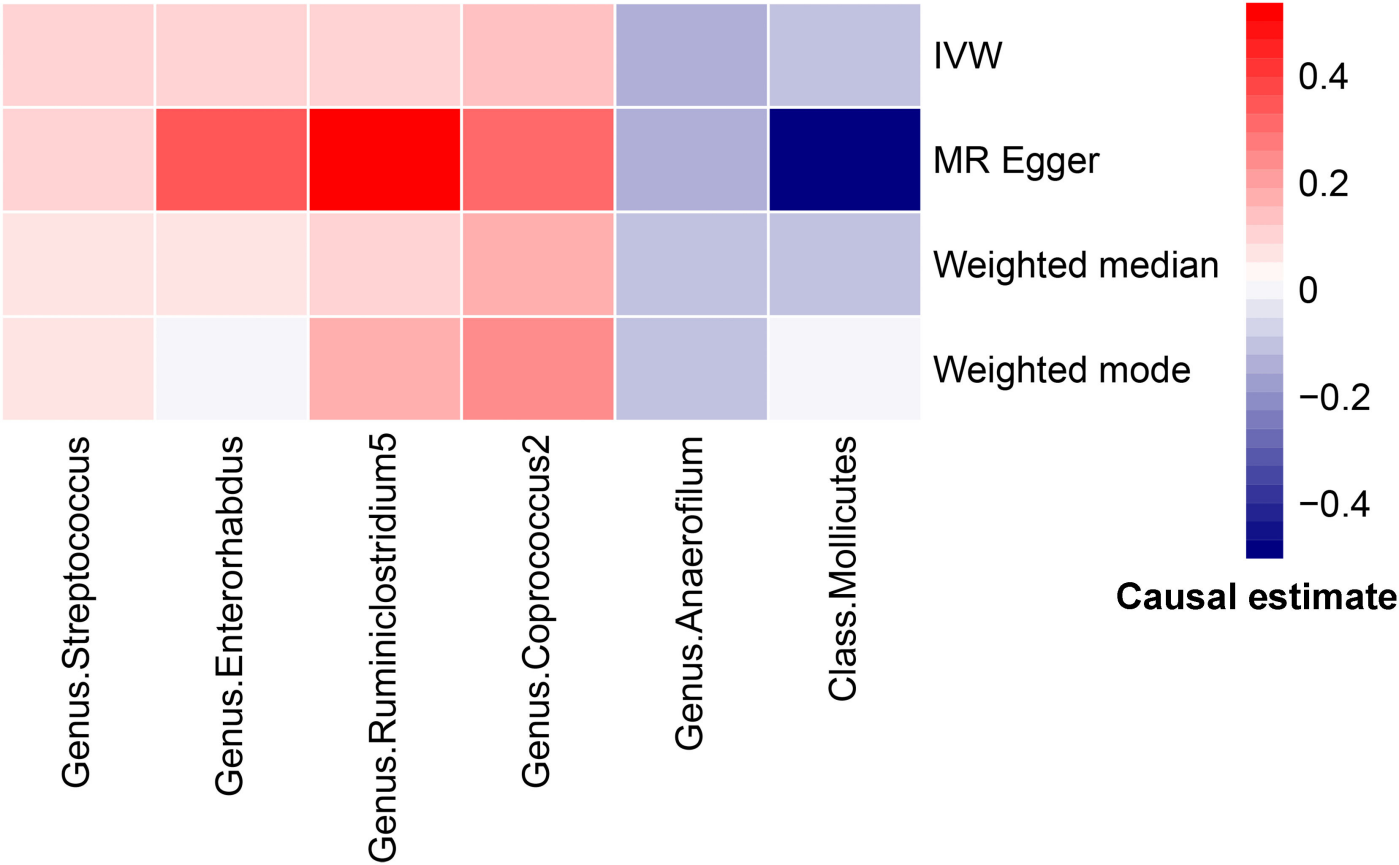
Heatmap for MR analysis of gut microbiota and PCOS (causality estimates (SD or log(OR)) per 1-SD increment in gut microbiota features).

IVW result suggested that an increased SD in class *Mollicutes* abundance was causally associated with the risk of some PCOS features (OR = 1.118, 95% CI = 1.030–1.215, *P* = 0.008). For the gut microbiota genus classification, one SD higher in *Anaerofilum* (OR = 1.090; 95% CI = 1.025–1.160; *P* = 0.006), *Coprococcus2* (OR = 1.119; 95% CI = 1.06–1.244; *P* = 0.039), *Ruminiclostridium5* (OR = 1.168; 95% CI = 1.049–1.302; *P* = 0.005) increased the risk of certain PCOS features. We identified positive, potentially causal associations between *Enterorhabdus* (OR = 0.867; 95% CI = 0.786–0.957; *P* = 0.005), *Streptococcus* (OR = 0.887; 95% CI = 0.800–0.982; *P* = 0.021) and some PCOS features. The results were consistent across all MR analysis methods (**Figures 3**, **4**). However, none of the gut microbiota was related to PCOS features after Bonferroni correction (*P* < 2.55 × 10^-4^).

### 3.3 Sensitivity analysis

**Figure 5:**
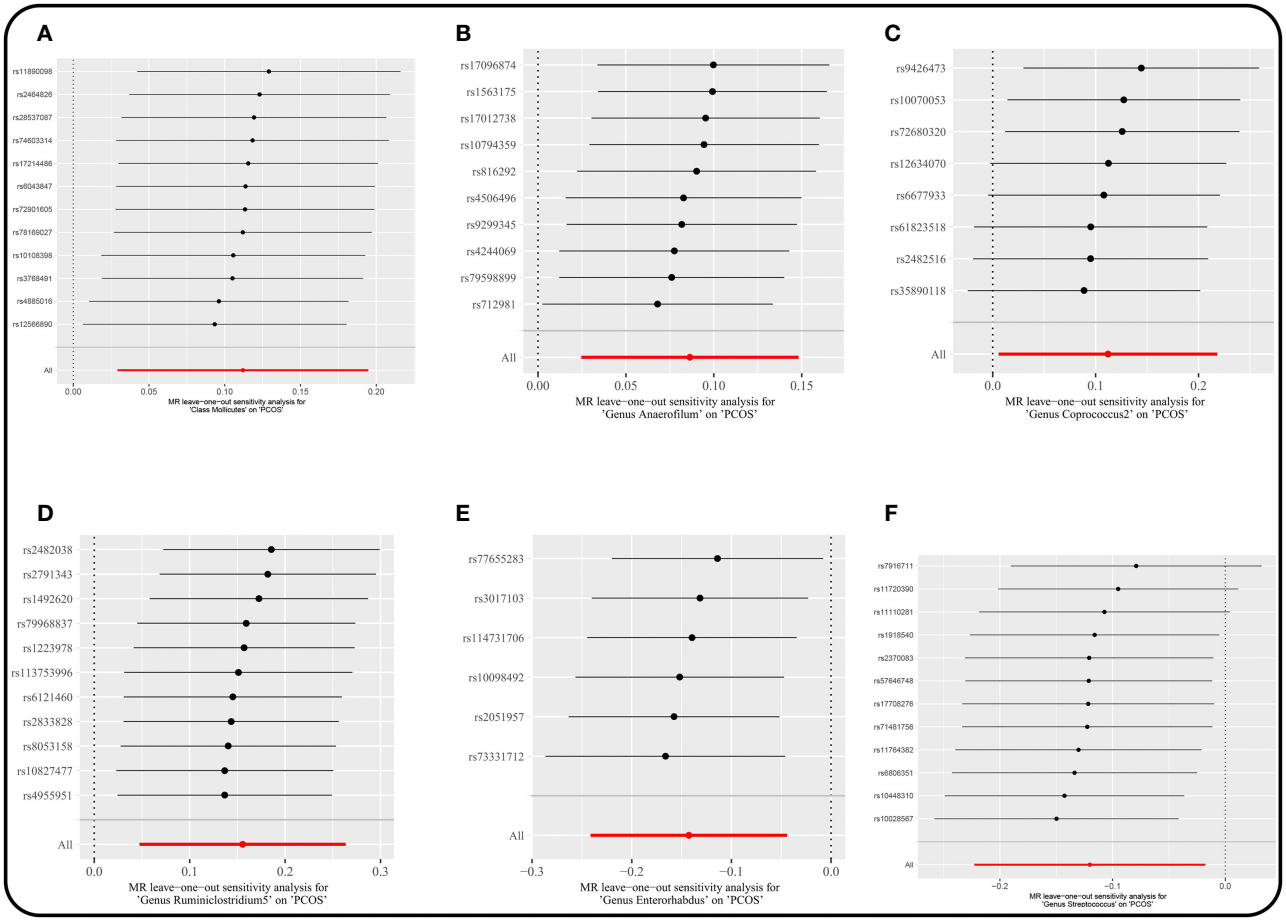
Leave-one-out plots for the causal association between gut microbiota and PCOS. (locus-wide significance, P<1×10^-5^). **(A)** Class *Mollicutes*. **(B)** Genus *Anaerofilum*. **(C)** Genus *Coprococcus*2. **(D)** Genus *Ruminiclostridium*5. **(E)** Genus *Enterorhabdus*. **(F)** Genus *Streptococcus*.

There were no horizontal pleiotropy, heterogeneity, or outliers according to MR-Egger regression, Cochran’s *Q* test, and MR-PRESSO global test for significant causality of IVW results (**Supplementary Table 5**). MR Steiger filtering test for the direction of causality (gut microbiota as the exposure, PCOS as the outcome) was correct. In addition, the “leave-one-out” test confirmed the stability of the results (**Figure 5**).

**Figure 6:**
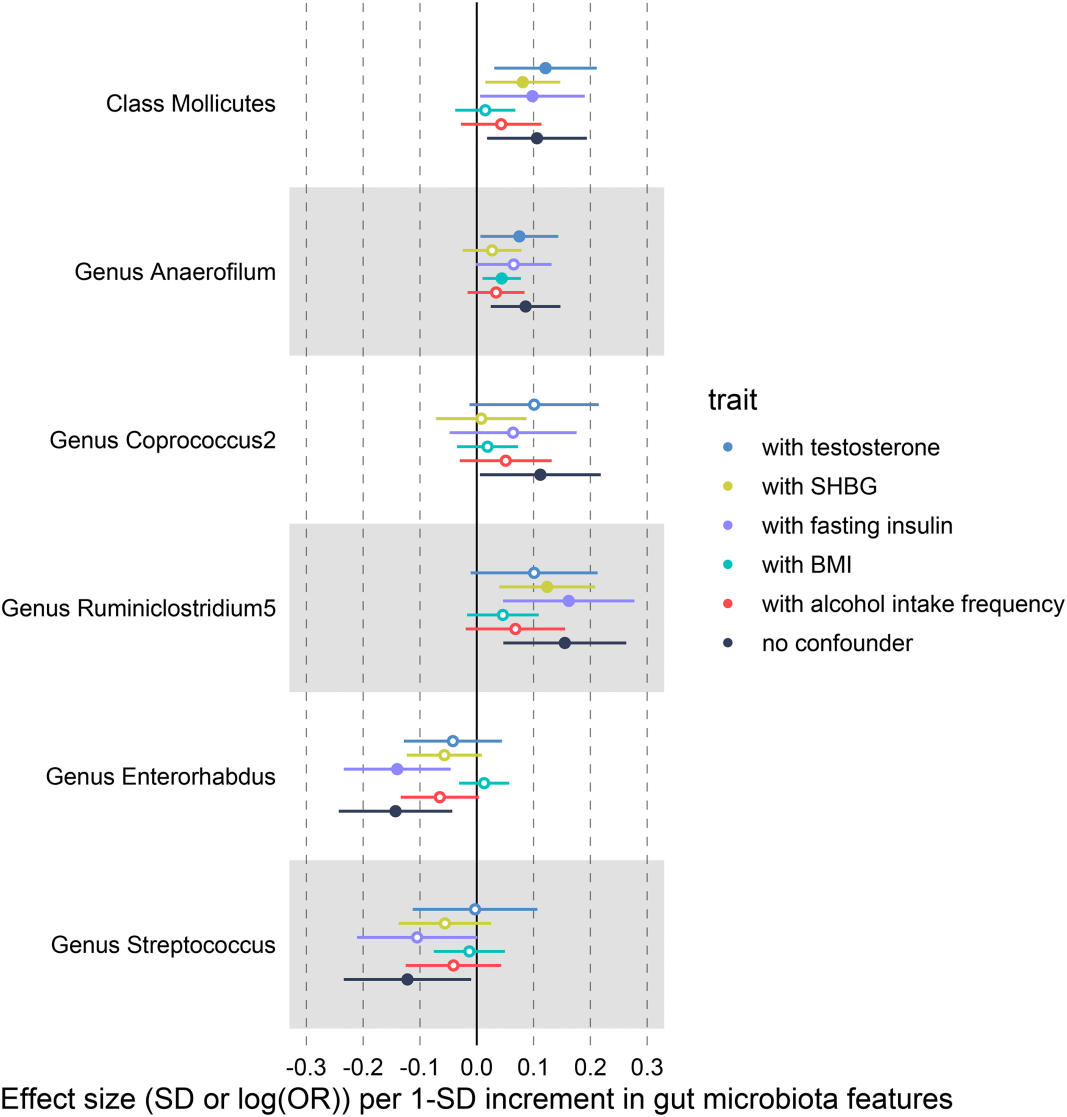
IVW-MR results before and after adjusting for SHBG, BMI and alcohol intake frequency using multivariate MR.

We conducted multivariable MR analyses of potential causality, with BMI, alcohol intake frequency, SHBG, total testosterone, and fasting insulin included as confounders. After correcting these confounders, the results indicated that all causal effects were reduced to varying degrees (**Figure 6**, **Supplementary Data S6**). For example, the causal effect of *Mollicutes* (OR = 1.015; *P* = 0.580) and *Enterorhabdus* (OR = 1.013; *P* = 0.564) on PCOS features approached null when BMI was included in the analysis. Especially *Streptococcus* was not causally associated with PCOS after adjusting for the above confounders separately. In addition, the causal effect of *Coprococcus2* on PCOS features reduced to null (OR = 1.008; *P* = 0.844) after adding SHBG as a covariate, and after adding alcohol intake frequency, the causal effect of *Anaerofilum* decreased to invalid (OR = 1.035; *P* = 0.184).

### 3.4 MR analysis (genome-wide significance, *P*<1×10^-8^)

We extracted SNPs significantly associated with gut microbiota for MR analysis (**Supplementary Data S7**). The results showed that elevated phylum *Actinobacteria* and class *Actinobacteria* reduced the risk of some PCOS features (**Supplementary Table 4**). However, sensitivity analyses could not be performed (only one SNP), and these results should be considered cautiously due to the possibility of spurious associations cannot be excluded.

### 3.5 Reverse MR analysis

A total of 31 SNPs (*P* < 1×10^-5^) associated with PCOS features were used for reverse MR analysis (**Supplementary Data S3**). **Supplementary Data S8** presented all MR results for associations of PCOS features on gut microbiota. Thirteen causal associations passed the nominal p-value significance threshold 0.05, but the PCOS features were not associated with any gut microbiota after multiple testing corrections (**Supplementary Table 6**). Sensitivity analysis suggested certain heterogeneity, horizontal pleiotropy, and outliers. Steiger filtering test for the direction of causality was correct (**Supplementary Table 7**).

According to the SNP selection threshold (*P* < 5×10^-8^), only rs74485684 was the effective IV. Reverse MR analysis suggested that 13 causal relationships were statistically significant (**Supplementary Data S9**). However, PCOS features were not associated with gut microbiota after Bonferroni correction. The number of IVs was too insufficient for sensitivity analyses.

### 3.6 Replication MR analysis

In the forward replication analysis, 21 SNPs were included with a threshold of *P* < 5×10^-8^. The Wald ratio method suggested no causal relationship between gut microbiota and PCOS features. We extracted 92 qualified SNPs (*P* < 1×10^-5^) for MR analysis. The results indicated protective causalities between 6 gut microbiota and PCOS features; two gut microbiota were risk factors for PCOS features. However, none of them were statistically significant after the Bonferroni correction (*P* < 2.55×10^-4^) (**Supplementary Table 8**).

In reverse replication analysis, no SNPs were extracted with a threshold of *P* < 5×10^-8^. We analyzed 14 eligible SNPs (*P* < 1×10^-5^) for MR analysis. The results indicated that PCOS was causally associated with five gut microbiota taxa but not after Bonferroni correction (**Supplementary Table 9**).

## 4 Discussion

We assessed causal associations between 196 gut microbiota taxa and PCOS features using bidirectional two-sample MR. With the SNP selection threshold at the level of *P* < 1×10^-5^, we found evidence of causal associations between 6 microbiota taxa and certain PCOS features. However, the causal effect did not reach statistical significance after the Bonferroni correction. Furthermore, the causal effects decreased considerably after including obesity (BMI), alcohol intake frequency, SHBG, hyperandrogenemia, and fasting insulin in the multivariable MR analysis. Reverse MR and analyses based on extracted SNP thresholds of *P* < 5×10^-8^ revealed no causal associations between gut microbiota and PCOS features due to the limited SNPs and unsupported sensitivity analysis. The results of the present study suggest that the earlier reported associations between them could be caused by biases such as confounders or reverse causation. Gut microbiota is likely not an independent cause of PCOS. Instead, the effect is mediated by multiple factors (i.e., BMI, SHBG, testosterone, and alcohol intake frequency).

A prospective cohort study including 102 PCOS and 201 controls matched for age and BMI suggested that gut microbial profiles did not differ significantly between PCOS and non-PCOS women (46). Another previous work suggested that no clear evidence of difference in gut microbiota between PCOS patients and healthy controls was retrieved after clinical practice (47). These findings were consistent with our study.

However, a cohort study supported the association of gut microbiota with PCOS (48). Notably, the outcomes likely reflect the smaller sample size (37 PCOS; 21 controls) and for specific subjects (obese adolescents). Therefore, it merits some caution in interpretation. The causal relationship between gut microbiota and PCOS was proposed by a recent MR study (49). In contrast, our study does not support this causal relationship. Above all, this previous data was analyzed by extracting gut microbiota-associated SNPs at the threshold of *P* < 1×10^-5^. This could potentially contradict the MR assumption that IVs must be strongly correlated with exposure. Secondly, the variance was not thoroughly evaluated, which could explain the stronger effects observed. In our MR analysis showed that SNPs explain only a minor portion of gut microbiota traits (*R* ^2^: 0.89%–1.91%). Furthermore, the bias of results and conclusions may be the consequence of un-corrected confounders (e.g., body mass index, alcohol consumption). Thus there could be spurious causal associations between gut microbiota and PCOS.

Since gut microbiota is susceptible to individual diet, sex, BMI, alcohol and drug intake. Earlier studies have shown that higher BMI is strongly associated with altered gut microbiota, and obesity is associated with elevated androgens and insulin resistance in PCOS (50, 51). Therefore, gut microbiota could influence the progression of PCOS through obesity and related mediators. Gu and colleagues showed significantly greater efficacy for weight control and lifestyle modifications in subjects with PCOS experiencing metabolic dysfunction and reproductive disorders in a meta-analysis including ten randomized controlled trials (52). Some studies may overestimate the effect size when the confounding factors were not undertaken in the calculation. These confounding factors could affect the veracity and robustness of the results.

The strengths of our study include the use of the largest GWAS dataset for bidirectional two-sample MR analysis. We extracted exposure-associated SNPs from two different threshold levels for analysis to obtain comprehensive and potentially causal relationships between gut microbiota and PCOS features. In addition, multiple sensitivity analyses are conducted to ensure the stability of the results.

However, our study has several limitations. We analyzed the genetic database based on European ancestry, which may not adapt to other ancestry populations. Further studies with other populations are required to confirm the generalizability of the results. The LDSC analyses reported a weak genetic correlation between gut microbiota on PCOS and small variance estimates. Furthermore, PCOS is a complex endocrine disease with diverse clinical features (e.g., obesity, hyperandrogenemia, infertility, and type 2 diabetes mellitus). We could not access the clinical phenotypes of individuals to perform subgroup analyses to investigate the association of the gut microbiota with different phenotypes of PCOS in GWAS databases. Thus, we explored the genetic association of gut microbiota with the PCOS features provided in databases. More precise and comprehensive genome-wide data on PCOS are warranted to explore the association of gut microbiota with different PCOS phenotypes.

## 5 Conclusions

Our MR analyses did not support the causal relationships between the gut microbiota and PCOS features at the genetic level. SNPs of the gut microbiota only explain a small portion of the pathogenesis of PCOS features. Large-scale gut microbiota and PCOS GWAS studies are warranted to clarify the genetic association.

## Data availability statement

The original contributions presented in the study are included in the article/**Supplementary Material**. Further inquiries can be directed to the corresponding author.

## Author contributions

JW: Conceptualization, Methodology, Software, Data curation, Writing – original draft. PF: Conceptualization, Writing – review & editing, Validation. GC: Conceptualization, Validation, Writing – review & editing. CC: Validation, Writing – review & editing. ZC: Writing – review & editing, Conceptualization, Methodology, Software, Supervision.
