# Supplementary material for: Gut microbiota and polycystic ovary syndrome, focus on genetic associations: a bidirectional Mendelian randomization study
Source: Front Endocrinol (Lausanne). 2024 Jan 22;15:1275419. doi: 10.3389/fendo.2024.1275419 (PMC10838976; doi:10.3389/fendo.2024.1275419)
Supplement: Supplementary file 1 [file DataSheet_1.zip › Supplementary Material/Table S2.DOCX]

| **TABLE S2.** Characteristics and details of the phenotypes analyzed in the study. | | | | | |
| --- | --- | --- | --- | --- | --- |
| **Phenotype** | **Type of trait** | **GWAS reference** | **Consortium** | **Unit** | **Sample size** |
| Gut microbiota | Continuous | Kurilshikov et al, 2021 | MiBioGen | SD | 18,340 |
| PCOS | Binary | NA | FinnGen R8 | log odds | 27,943 cases  162,936 controls |
| Replication PCOS | Binary | Day F et al, 2018 | NA | log odds | 10,074 cases 103,164 controls |
| Body mass index | Continuous | Ben Elsworth et al, 2018 | MRC-IEU | SD | 461,460 |
| Alcohol intake frequency | Categorical Ordered | Ben Elsworth et al, 2018 | MRC-IEU | SD | 462,346 |
| SHBG | Continuous | Richmond ea al, 2020 | UK Biobank | SD | 214,989 |
| Fasting insulin | Continuous | Lagou V et al, 2021 | MAGIC | pmol/L | 50,404 |
| Total testosterone | Continuous | Richmond et al, 2020 | MRC-IEU | SD | 199,569 |
| Abbreviations: PCOS: polycystic ovary syndrome; SHBG: sex hormone binding globulin; MAGIC: Meta-Analyses of Glucose and Insulin-related traits Consortium; SD: standard deviation ; NA: not available. | | | | | |
