# Supplementary material for: Gut microbiota and polycystic ovary syndrome, focus on genetic associations: a bidirectional Mendelian randomization study
Source: Front Endocrinol (Lausanne). 2024 Jan 22;15:1275419. doi: 10.3389/fendo.2024.1275419 (PMC10838976; doi:10.3389/fendo.2024.1275419)
Supplement: Supplementary file 1 [file DataSheet_1.zip › Supplementary Material/Table S3.DOCX]

| **TABLE S3.** Results of linkage disequilibrium score (LDSC) regression for the genetic correlations between gut microbiota and PCOS. | | | | | |
| --- | --- | --- | --- | --- | --- |
| **Gut microbiota (exposure)** | **Outcome** | **rg** | **SE** | ***P*-value** | ***R*^2^** |
| class *Mollicutes* | PCOS | -0.164 | 0.358 | 0.648 | 1.783% |
| genus *Anaerofilum* | PCOS | 0.724 | 0.321 | 0.024 | 1.584% |
| genus *Coprococcus2* | PCOS | -0.359 | 0.227 | 0.114 | 1.160% |
| genus *Ruminiclostridium5* | PCOS | NA | NA | NA | 1.648% |
| genus *Enterorhabdus* | PCOS | 0.374 | 0.289 | 0.196 | 0.888% |
| genus *Streptococcus* | PCOS | 0.022 | 0.246 | 0.929 | 1.911% |
| PCOS: polycystic ovary syndrome; SE: standard error; *R*^2^: proportion of variance in exposure variable explained by SNPs; NA: not available. | | | | | |
