# Supplementary material for: Gut microbiota and polycystic ovary syndrome, focus on genetic associations: a bidirectional Mendelian randomization study
Source: Front Endocrinol (Lausanne). 2024 Jan 22;15:1275419. doi: 10.3389/fendo.2024.1275419 (PMC10838976; doi:10.3389/fendo.2024.1275419)
Supplement: Supplementary file 1 [file DataSheet_1.zip › Supplementary Material/Table S4.DOCX]

| **TABLE S4.** MR analysis results of the causal relationship between gut microbiota and PCOS risk | | | | | | |
| --- | --- | --- | --- | --- | --- | --- |
| **Exposure** | **Outcome** | **N.SNP** | ***F*** | **Method** | **OR (95%CI)** | ***P*-value** |
| locus-wide significance, *P* < 1×10^-5^ | | | | | | |
| Class *Mollicutes* | PCOS | 12 | 21.29 | IVW | 1.118 (1.030-1.215) | 0.008 |
|  |  | 12 | 21.29 | MR Egger | 1.105 (0.835-1.464) | 0.500 |
|  |  | 12 | 21.29 | Weighted median | 1.086 (0.972-1.213) | 0.147 |
|  |  | 12 | 21.29 | Weighted mode | 1.080 (0.918-1.272) | 0.373 |
| Genus *Anaerofilum* | PCOS | 10 | 22.70 | IVW | 1.090 (1.025-1.160) | 0.006 |
|  |  | 10 | 22.70 | MR Egger | 1.393 (0.995-1.948) | 0.089 |
|  |  | 10 | 22.70 | Weighted median | 1.060 (0.971-1.156) | 0.194 |
|  |  | 10 | 22.70 | Weighted mode | 1.014 (0.889-1.155) | 0.843 |
| Genus *Coprococcus2* | PCOS | 8 | 20.77 | IVW | 1.119 (1.006-1.244) | 0.039 |
|  |  | 8 | 20.77 | MR Egger | 1.709 (0.739-3.950) | 0.257 |
|  |  | 8 | 20.77 | Weighted median | 1.125 (0.974-1.298) | 0.109 |
|  |  | 8 | 20.77 | Weighted mode | 1.182 (0.945-1.477) | 0.186 |
| Genus *Ruminiclostridium5* | PCOS | 11 | 21.46 | IVW | 1.168 (1.049-1.302) | 0.005 |
|  |  | 11 | 21.46 | MR Egger | 1.360 (0.865-2.138) | 0.216 |
|  |  | 11 | 21.46 | Weighted median | 1.187 (1.025-1.374) | 0.022 |
|  |  | 11 | 21.46 | Weighted mode | 1.278 (0.964-1.693) | 0.119 |
| Genus *Enterorhabdus* | PCOS | 6 | 21.21 | IVW | 0.867 (0.786-0.957) | 0.005 |
|  |  | 6 | 21.21 | MR Egger | 0.883 (0.681-1.144) | 0.399 |
|  |  | 6 | 21.21 | Weighted median | 0.890 (0.780-1.017) | 0.087 |
|  |  | 6 | 21.21 | Weighted mode | 0.909 (0.775-1.066) | 0.292 |
| Genus *Streptococcus* | PCOS | 12 | 22.82 | IVW | 0.887 (0.800-0.982) | 0.021 |
|  |  | 12 | 22.82 | MR Egger | 0.605 (0.414-0.883) | 0.026 |
|  |  | 12 | 22.82 | Weighted median | 0.904 (0.782-1.045) | 0.172 |
|  |  | 12 | 22.82 | Weighted mode | 0.913 (0.680-1.228) | 0.561 |
| Genome-wide statistical significance, *P* < 5×10^-8^ | | | | | | |
| Phylum *Actinobacteria* | PCOS | 1 | 58.16 | Wald ratio | 0.676 (0.545-0.838) | 3.68E-04 |
| Class *Actinobacteria* | PCOS | 1 | 85.38 | Wald ratio | 0.719 (0.608-0.851) | 1.24E-04 |
| PCOS: Polycystic Ovary Syndrome; N.SNP: number of single nucleotide polymorphis; MR: Mendelian randomization; IVW: Inverse variance weighted; *F*: mean of F-statistic; OR: odds ratio; CI: confidence interval. | | | | | | |
