# Supplementary material for: Gut microbiota and polycystic ovary syndrome, focus on genetic associations: a bidirectional Mendelian randomization study
Source: Front Endocrinol (Lausanne). 2024 Jan 22;15:1275419. doi: 10.3389/fendo.2024.1275419 (PMC10838976; doi:10.3389/fendo.2024.1275419)
Supplement: Supplementary file 1 [file DataSheet_1.zip › Supplementary Material/Table S5.DOCX]

| **TABLE S5.** Sensitivity analysis and MR Steiger directionality test of gut microbiota on PCOS. | | | | | | | | |
| --- | --- | --- | --- | --- | --- | --- | --- | --- |
| **Exposure** | **Outcome** | **Heterogeneity** | | **Horizontal pleiotropy** | | | **MR Steiger** | |
|  |  | **Cochran’s *Q*** | ***P*** | **Egger intercept** | ***P*^1^** | **MR-PRESSO^2^** | **direction^3^** | ***P*** |
| class Mollicutes | PCOS | 6.814 | 0.814 | 0.001 | 0.933 | 0.822 | TRUE | 7.26E-28 |
| genus Anaerofilum | PCOS | 8.592 | 0.476 | -0.027 | 0.184 | 0.510 | TRUE | 3.64E-23 |
| genus Coprococcus2 | PCOS | 5.323 | 0.621 | -0.031 | 0.356 | 0.651 | TRUE | 2.25E-18 |
| genus Ruminiclostridium5 | PCOS | 9.504 | 0.485 | -0.009 | 0.515 | 0.493 | TRUE | 6.11E-24 |
| genus Enterorhabdus | PCOS | 3.083 | 0.687 | -0.003 | 0.890 | 0.725 | TRUE | 5.84E-13 |
| genus Streptococcus | PCOS | 11.086 | 0.436 | 0.032 | 0.067 | 0.419 | TRUE | 1.69E-28 |
| ^1^The p-values of MR-egger intercept test. ^2^The p-values of MR-PRESSO global test. ^3^The correct direction of causality is from gut microbiota to PCOS, which is presented as TRUE. MR: Mendelian randomization; PCOS: Polycystic Ovary Syndrome; MR-PRESSO: MR-Pleiotropy Residual Sum and Outlier. | | | | | | | | |
