# Supplementary material for: Gut microbiota and polycystic ovary syndrome, focus on genetic associations: a bidirectional Mendelian randomization study
Source: Front Endocrinol (Lausanne). 2024 Jan 22;15:1275419. doi: 10.3389/fendo.2024.1275419 (PMC10838976; doi:10.3389/fendo.2024.1275419)
Supplement: Supplementary file 1 [file DataSheet_1.zip › Supplementary Material/Table S6.DOCX]

| **TABLE S6.** Reverse MR analysis of the causal association between PCOS and gut microbiota (locus-wide significance, *P* < 1.0×10^-5^). | | | | | | |
| --- | --- | --- | --- | --- | --- | --- |
| **Exposure** | **Outcome** | **N.SNP** | ***F*** | **Method** | **OR (95%CI)** | ***P*-value** |
| PCOS | phylum Actinobacteria | 31 | 23.29 | IVW | 0.909 (0.830-0.995) | 0.038 |
|  |  | 31 | 23.29 | MR Egger | 0.791 (0.564-1.108) | 0.184 |
|  |  | 31 | 23.29 | Weighted median | 0.915 (0.808-1.036) | 0.161 |
|  |  | 31 | 23.29 | Weighted mode | 0.932 (0.736-1.180) | 0.561 |
| PCOS | class Actinobacteria | 31 | 23.29 | IVW | 0.873 (0.786-0.970) | 0.012 |
|  |  | 31 | 23.36 | MR Egger | 0.568 (0.396-0.815) | 0.005 |
|  |  | 31 | 23.36 | Weighted median | 0.902 (0.789-1.031) | 0.130 |
|  |  | 31 | 23.36 | Weighted mode | 0.965 (0.708-1.312) | 0.820 |
| PCOS | order Bifidobacteriales | 31 | 23.29 | IVW | 0.881 (0.780-0.994) | 0.039 |
|  |  | 31 | 23.36 | MR Egger | 0.543 (0.358-0.824) | 0.008 |
|  |  | 31 | 23.36 | Weighted median | 0.911 (0.788-1.054) | 0.212 |
|  |  | 31 | 23.36 | Weighted mode | 0.926 (0.673-1.274) | 0.640 |
| PCOS | family Bacteroidaceae | 31 | 23.29 | IVW | 1.093 (1.004-1.191) | 0.041 |
|  |  | 31 | 23.36 | MR Egger | 1.249 (0.911-1.714) | 0.178 |
|  |  | 31 | 23.36 | Weighted median | 1.072 (0.948-1.214) | 0.268 |
|  |  | 31 | 23.36 | Weighted mode | 1.068 (0.860-1.328) | 0.555 |
| PCOS | family Bifidobacteriaceae | 31 | 23.29 | IVW | 0.881 (0.780-0.994) | 0.039 |
|  |  | 31 | 23.36 | MR Egger | 0.543 (0.358-0.824) | 0.008 |
|  |  | 31 | 23.36 | Weighted median | 0.911 (0.788-1.054) | 0.211 |
|  |  | 31 | 23.36 | Weighted mode | 0.926 (0.672-1.276) | 0.642 |
| PCOS | genus Bacteroides | 31 | 23.29 | IVW | 1.093 (1.004-1.191) | 0.041 |
|  |  | 31 | 23.36 | MR Egger | 1.249 (0.911-1.714) | 0.178 |
|  |  | 31 | 23.36 | Weighted median | 1.072 (0.949-1.211) | 0.260 |
|  |  | 31 | 23.36 | Weighted mode | 1.068 (0.850-1.343) | 0.575 |
| PCOS | genus Barnesiella | 31 | 23.29 | IVW | 1.108 (1.001-1.226) | 0.048 |
|  |  | 31 | 23.36 | MR Egger | 0.941 (0.644-1.375) | 0.757 |
|  |  | 31 | 23.36 | Weighted median | 1.103 (0.958-1.270) | 0.171 |
|  |  | 31 | 23.36 | Weighted mode | 1.145 (0.867-1.513) | 0.347 |
| PCOS | genus Bifidobacterium | 31 | 23.29 | IVW | 0.877 (0.766-0.991) | 0.035 |
|  |  | 31 | 23.36 | MR Egger | 0.516 (0.342-0.780) | 0.004 |
|  |  | 31 | 23.36 | Weighted median | 0.965 (0.836-1.115) | 0.631 |
|  |  | 31 | 23.36 | Weighted mode | 0.959 (0.693-1.327) | 0.803 |
| PCOS | genus Christensenellaceae R 7group | 31 | 23.29 | IVW | 0.899 (0.821-0.984) | 0.021 |
|  |  | 31 | 23.36 | MR Egger | 0.634 (0.454-0.887) | 0.012 |
|  |  | 31 | 23.36 | Weighted median | 0.905 (0.794-1.032) | 0.137 |
|  |  | 31 | 23.36 | Weighted mode | 0.864 (0.679-1.101) | 0.247 |
| PCOS | genus Erysipelotrichaceae UCG003 | 5 | 20.47 | IVW | 1.299 (1.006-1.677) | 0.045 |
|  |  | 5 |  | MR Egger | 2.083 (0.723-6.003) | 0.267 |
|  |  | 5 |  | Weighted median | 1.294 (0.923-1.814) | 0.134 |
|  |  | 5 |  | Weighted mode | 1.301 (0.817-2.071) | 0.330 |
| PCOS | genus Ruminococcaceae UCG004 | 30 | 23.36 | IVW | 0.844 (0.749-0.951) | 0.005 |
|  |  | 30 | 23.36 | MR Egger | 0.887 (0.563-1.396) | 0.608 |
|  |  | 30 | 23.36 | Weighted median | 0.852 (0.722-1.006) | 0.059 |
|  |  | 30 | 23.36 | Weighted mode | 0.841 (0.605-1.170) | 0.312 |
| PCOS | genus Ruminococcus gnavus group | 30 | 23.36 | IVW | 1.211 (1.047-1.401) | 0.010 |
|  |  | 30 | 23.36 | MR Egger | 2.019 (1.163-3.508) | 0.019 |
|  |  | 30 | 23.36 | Weighted median | 1.271 (1.034-1.562) | 0.023 |
|  |  | 30 | 23.36 | Weighted mode | 1.409 (0.905-2.194) | 0.139 |
| PCOS | genus Veillonella | 30 | 23.36 | IVW | 1.150 (1.002-1.319) | 0.047 |
|  |  | 30 | 23.36 | MR Egger | 0.914 (0.543-1.537) | 0.737 |
|  |  | 30 | 23.36 | Weighted median | 1.132 (0.944-1.357) | 0.180 |
|  |  | 30 | 23.36 | Weighted mode | 0.984 (0.670-1.444) | 0.934 |
| PCOS: Polycystic Ovary Syndrome; N.SNP: number of single nucleotide polymorphis; MR: Mendelian randomization; IVW: Inverse variance weighted; *F*: mean of F-statistic; OR: odds ratio; CI: confidence interval. | | | | | | |
