# Supplementary material for: Gut microbiota and polycystic ovary syndrome, focus on genetic associations: a bidirectional Mendelian randomization study
Source: Front Endocrinol (Lausanne). 2024 Jan 22;15:1275419. doi: 10.3389/fendo.2024.1275419 (PMC10838976; doi:10.3389/fendo.2024.1275419)
Supplement: Supplementary file 1 [file DataSheet_1.zip › Supplementary Material/Table S7.DOCX]

| **TABLE S7.** Sensitivity analysis and MR Steiger directionality test of PCOS on gut microbiota. | | | | | | | | |
| --- | --- | --- | --- | --- | --- | --- | --- | --- |
| **Exposure** | **Outcome** | **Heterogeneity** | | **Horizontal pleiotropy** | | | **MR Steiger** | |
|  |  | **Cochran’s *Q*** | ***P*** | **Egger intercept** | ***P*^1^** | **MR-PRESSO^2^** | **Direction^3^** | ***P*** |
| PCOS | phylum Actinobacteria | 33.23 | 0.313 | 0.008 | 0.409 | 0.320 | TRUE | 2.85E-27 |
| PCOS | class Actinobacteria | 40.429 | 0.097 | 0.025 | 0.022 | 0.104 | TRUE | 2.68E-24 |
| PCOS | order Bifidobacteriales | 49.021 | 0.016 | 0.028 | 0.025 | 0.010 | TRUE | 1.46E-22 |
| PCOS | family Bacteroidaceae | 23.239 | 0.805 | -0.008 | 0.397 | 0.816 | TRUE | 7.48E-31 |
| PCOS | family Bifidobacteriaceae | 49.021 | 0.016 | 0.028 | 0.025 | 0.014 | TRUE | 1.46E-22 |
| PCOS | genus Bacteroides | 23.239 | 0.805 | -0.008 | 0.397 | 0.815 | TRUE | 7.48E-31 |
| PCOS | genus Barnesiella | 32.846 | 0.329 | 0.009 | 0.389 | 0.328 | TRUE | 2.77E-27 |
| PCOS | genus Bifidobacterium | 49.437 | 0.014 | 0.031 | 0.014 | 0.012 | TRUE | 2.02E-22 |
| PCOS | genus Christensenellaceae R 7group | 23.382 | 0.799 | 0.020 | 0.043 | 0.793 | TRUE | 1.78E-30 |
| PCOS | genus Erysipelotrichaceae UCG003 | 2.087 | 0.720 | -0.026 | 0.434 | 0.722 | TRUE | 1.17E-04 |
| PCOS | genus Ruminococcaceae UCG004 | 25.926 | 0.629 | -0.003 | 0.826 | 0.636 | TRUE | 3.68E-27 |
| PCOS | genus Ruminococcus gnavus group | 23.826 | 0.737 | -0.029 | 0.070 | 0.734 | TRUE | 3.88E-28 |
| PCOS | genus Veillonella | 37.378 | 0.137 | 0.013 | 0.377 | 0.133 | TRUE | 1.27E-24 |
| ^1^The p-values of MR-egger intercept test. ^2^The p-values of MR-PRESSO global test. ^3^The correct direction of causality is from gut microbiota to PCOS, which is presented as TRUE. MR: Mendelian randomization; PCOS: Polycystic Ovary Syndrome; MR-PRESSO: MR-Pleiotropy Residual Sum and Outlier. | | | | | | | | |
