# Supplementary material for: Gut microbiota and polycystic ovary syndrome, focus on genetic associations: a bidirectional Mendelian randomization study
Source: Front Endocrinol (Lausanne). 2024 Jan 22;15:1275419. doi: 10.3389/fendo.2024.1275419 (PMC10838976; doi:10.3389/fendo.2024.1275419)
Supplement: Supplementary file 1 [file DataSheet_1.zip › Supplementary Material/Table S8.DOCX]

| **TABLE S8.** Replication MR analysis results of the causal relationship between gut microbiota and PCOS risk. | | | | | | |
| --- | --- | --- | --- | --- | --- | --- |
| **Exposure** | **Outcome** | **N.SNP** | ***F*** | **Method** | **OR (95%CI)** | ***P*-value** |
| locus-wide significance, *P*< 1×10^-5^ | | | | | | |
| Class *Clostridia* | PCOS | 13 | 22.02 | IVW | 0.645 (0.441-0.941) | 0.023 |
|  |  | 13 | 22.02 | MR Egger | 0.838 (0.333-2.110) | 0.715 |
|  |  | 13 | 22.02 | Weighted median | 0.719 (0.447-1.157) | 0.174 |
|  |  | 13 | 22.02 | Weighted mode | 0.716 (0.406-1.264) | 0.272 |
| Order *Clostridiales* | PCOS | 14 | 21.72 | IVW | 0.633 (0.444-0.902) | 0.011 |
|  |  | 14 | 21.72 | MR Egger | 0.870 (0.366-2.063) | 0.757 |
|  |  | 14 | 21.72 | Weighted median | 0.716 (0.448-1.145) | 0.163 |
|  |  | 14 | 21.72 | Weighted mode | 0.699 (0.416-1.173) | 0.198 |
| Family *Streptococcaceae* | PCOS | 13 | 22.57 | IVW | 0.668 (0.482-0.925) | 0.015 |
|  |  | 13 | 22.57 | MR Egger | 0.423 (0.113-1.586) | 0.228 |
|  |  | 13 | 22.57 | Weighted median | 0.656 (0.421-1.021) | 0.062 |
|  |  | 13 | 22.57 | Weighted mode | 0.632 (0.277-1.443) | 0.297 |
| Genus *Actinomyces* | PCOS | 7 | 21.04 | IVW | 0.730 (0.548-0.973) | 0.032 |
|  |  | 7 | 21.04 | MR Egger | 0.706 (0.326-1.533) | 0.420 |
|  |  | 7 | 21.04 | Weighted median | 0.674 (0.466-0.976) | 0.037 |
|  |  | 7 | 21.04 | Weighted mode | 0.582 (0.314-1.079) | 0.136 |
| Genus *Candidatus Soleaferrea* | PCOS | 9 | 21.30 | IVW | 1.297 (1.011-1.664) | 0.041 |
|  |  | 9 | 21.30 | MR Egger | 7.185 (0.503-102.591) | 0.189 |
|  |  | 9 | 21.30 | Weighted median | 1.338 (0.962-1.861) | 0.084 |
|  |  | 9 | 21.30 | Weighted mode | 1.380 (0.814-2.339) | 0.266 |
| Genus *Ruminococcaceae UCG005* | PCOS | 14 | 21.21 | IVW | 0.721 (0.539-0.965) | 0.028 |
|  |  | 14 | 21.21 | MR Egger | 0.830 (0.367-1.880) | 0.664 |
|  |  | 14 | 21.21 | Weighted median | 0.753 (0.505-1.123) | 0.164 |
|  |  | 14 | 21.21 | Weighted mode | 0.816 (0.451-1.478) | 0.515 |
| Genus *Ruminococcaceae UCG011* | PCOS | 8 | 22.74 | IVW | 1.324 (1.053-1.667) | 0.017 |
|  |  | 8 | 22.74 | MR Egger | 3.089 (1.094-8.722) | 0.077 |
|  |  | 8 | 22.74 | Weighted median | 1.328 (1.003-1.759) | 0.047 |
|  |  | 8 | 22.74 | Weighted mode | 1.636 (0.964-2.775) | 0.111 |
| Genus *Streptococcus* | PCOS | 14 | 22.89 | IVW | 0.660 (0.484-0.900) | 0.009 |
|  |  | 14 | 22.89 | MR Egger | 0.428 (0.142-1.284) | 0.156 |
|  |  | 14 | 22.89 | Weighted median | 0.629 (0.418-0.947) | 0.026 |
|  |  | 14 | 22.89 | Weighted mode | 0.591 (0.288-1.215) | 0.176 |
| genome-wide statistical significance, *P*< 5×10^-8^ | | | | | | |
| Phylum Actinobacteria | PCOS | 1 | 58.16 | Wald ratio | 1.447 (0.612-3.417) | 0.340 |
| Class *Actinobacteria* | PCOS | 1 | 85.38 | Wald ratio | 1.419 (0.715-2.816) | 0.317 |
| Order *Bifidobacteriales* | PCOS | 2 | 57.64 | IVW | 1.282 (0.722-2.276) | 0.397 |
| Order *Gastranaerophilales* | PCOS | 1 | 30.01 | Wald ratio | 2.248 (1.251-4.039) | 0.007 |
| Family *Bifidobacteriaceae* | PCOS | 2 | 57.67 | IVW | 1.282 (0.722-2.276) | 0.397 |
| Family *Oxalobacteraceae* | PCOS | 1 | 29.82 | Wald ratio | 1.002 (0.571-1.758) | 0.993 |
| Family *Peptostreptococcaceae* | PCOS | 1 | 32.65 | Wald ratio | 0.859 (0.351-2.102) | 0.739 |
| Family *Streptococcaceae* | PCOS | 1 | 33.39 | Wald ratio | 0.490 (0.193-1.244) | 0.134 |
| Genus *Allisonella* | PCOS | 1 | 32.38 | Wald ratio | 0.899 (0.599-1.349) | 0.607 |
| Genus *Bifidobacterium* | PCOS | 2 | 59.89 | IVW | 1.276 (0.727-2.238) | 0.396 |
| Genus *Erysipelatoclostridium* | PCOS | 1 | 34.51 | Wald ratio | 1.167 (0.540-2.522) | 0.694 |
| Genus *Eubacterium* | PCOS | 1 | 32.79 | Wald ratio | 0.595 (0.202-1.750) | 0.345 |
| Genus *Oxalobacter* | PCOS | 1 | 31.22 | Wald ratio | 0.683 (0.401-1.162) | 0.160 |
| Genus *Romboutsia* | PCOS | 1 | 29.51 | Wald ratio | 0.860 (0.354-2.087) | 0.739 |
| Genus *Ruminococcaceae UCG013* | PCOS | 1 | 31.44 | Wald ratio | 0.643 (0.240-1.722) | 0.379 |
| Genus *Ruminococcus torques group* | PCOS | 1 | 31.52 | Wald ratio | 1.015 (0.364-2.828) | 0.978 |
| Genus *Streptococcus* | PCOS | 1 | 36.69 | Wald ratio | 0.509 (0.210-1.230) | 0.134 |
| Genus *Tyzzerella3* | PCOS | 1 | 35.46 | Wald ratio | 1.438 (0.831-2.489) | 0.194 |
| PCOS: Polycystic Ovary Syndrome; N.SNP: number of single nucleotide polymorphis; MR: Mendelian randomization; IVW: Inverse variance weighted; *F*: mean of F-statistic; OR: odds ratio; CI: confidence interval. | | | | | | |
