# Supplementary material for: Gut microbiota and polycystic ovary syndrome, focus on genetic associations: a bidirectional Mendelian randomization study
Source: Front Endocrinol (Lausanne). 2024 Jan 22;15:1275419. doi: 10.3389/fendo.2024.1275419 (PMC10838976; doi:10.3389/fendo.2024.1275419)
Supplement: Supplementary file 1 [file DataSheet_1.zip › Supplementary Material/Table S9.DOCX]

| **TABLE S9.** Replication MR analysis results of the causal relationship between PCOS and gut microbiota (*P*< 1×10^-5^). | | | | | | |
| --- | --- | --- | --- | --- | --- | --- |
| **Exposure** | **Outcome** | **N.SNP** | ***F*** | **Method** | **OR (95%CI)** | ***P*-value** |
| PCOS | Family *Defluviitaleaceae* | 13 | 22.19 | IVW | 0.945 (0.893-1.000) | 0.048 |
|  |  | 13 | 22.19 | MR Egger | 0.952 (0.747-1.215) | 0.702 |
|  |  | 13 | 22.19 | Weighted median | 0.958 (0.886-1.036) | 0.280 |
|  |  | 13 | 22.19 | Weighted mode | 0.962 (0.849-1.091) | 0.561 |
| PCOS | Genus *Collinsella* | 14 | 22.19 | IVW | 0.958 (0.919-0.999) | 0.047 |
|  |  | 14 | 22.19 | MR Egger | 0.964 (0.813-1.143) | 0.680 |
|  |  | 14 | 22.19 | Weighted median | 0.954 (0.899-1.011) | 0.113 |
|  |  | 14 | 22.19 | Weighted mode | 0.959 (0.866-1.061) | 0.429 |
| PCOS | Genus *Defluviitaleaceae UCG011* | 13 | 22.19 | IVW | 0.940 (0.888-0.925) | 0.033 |
|  |  | 13 | 22.19 | MR Egger | 0.931 (0.730-1.188) | 0.577 |
|  |  | 13 | 22.19 | Weighted median | 0.952 (0.882-1.028) | 0.210 |
|  |  | 13 | 22.19 | Weighted mode | 0.955 (0.839-1.008) | 0.503 |
| PCOS | Genus *Eubacterium oxidoreducens group* | 13 | 22.19 | IVW | 0.915 (0.850-0.985) | 0.018 |
|  |  | 13 | 22.19 | MR Egger | 0.933 (0.672-1.295) | 0.685 |
|  |  | 13 | 22.19 | Weighted median | 0.896 (0.808-0.993) | 0.036 |
|  |  | 13 | 22.19 | Weighted mode | 0.873 (0.716-1.064) | 0.873 |
| PCOS | Genus *Romboutsia* | 14 | 22.41 | IVW | 1.053 (1.010-1.099) | 0.016 |
|  |  | 14 | 22.41 | MR Egger | 1.044 (0.878-1.241) | 0.636 |
|  |  | 14 | 22.41 | Weighted median | 1.062 (1.001-1.127) | 0.047 |
|  |  | 14 | 22.41 | Weighted mode | 1.075 (0.968-1.193) | 0.200 |
| PCOS: Polycystic Ovary Syndrome; N.SNP: number of single nucleotide polymorphis; MR: Mendelian randomization; IVW: Inverse variance weighted; *F*: mean of F-statistic; OR: odds ratio; CI: confidence interval. | | | | | | |
